# Supplementary figures and images for: Kinetic modelling of sterol transport between plasma membrane and endo-lysosomes based on quantitative fluorescence and X-ray imaging data
Source: Front Cell Dev Biol. 2023 Oct 31;11:1144936. doi: 10.3389/fcell.2023.1144936 (PMC10644255; doi:10.3389/fcell.2023.1144936)

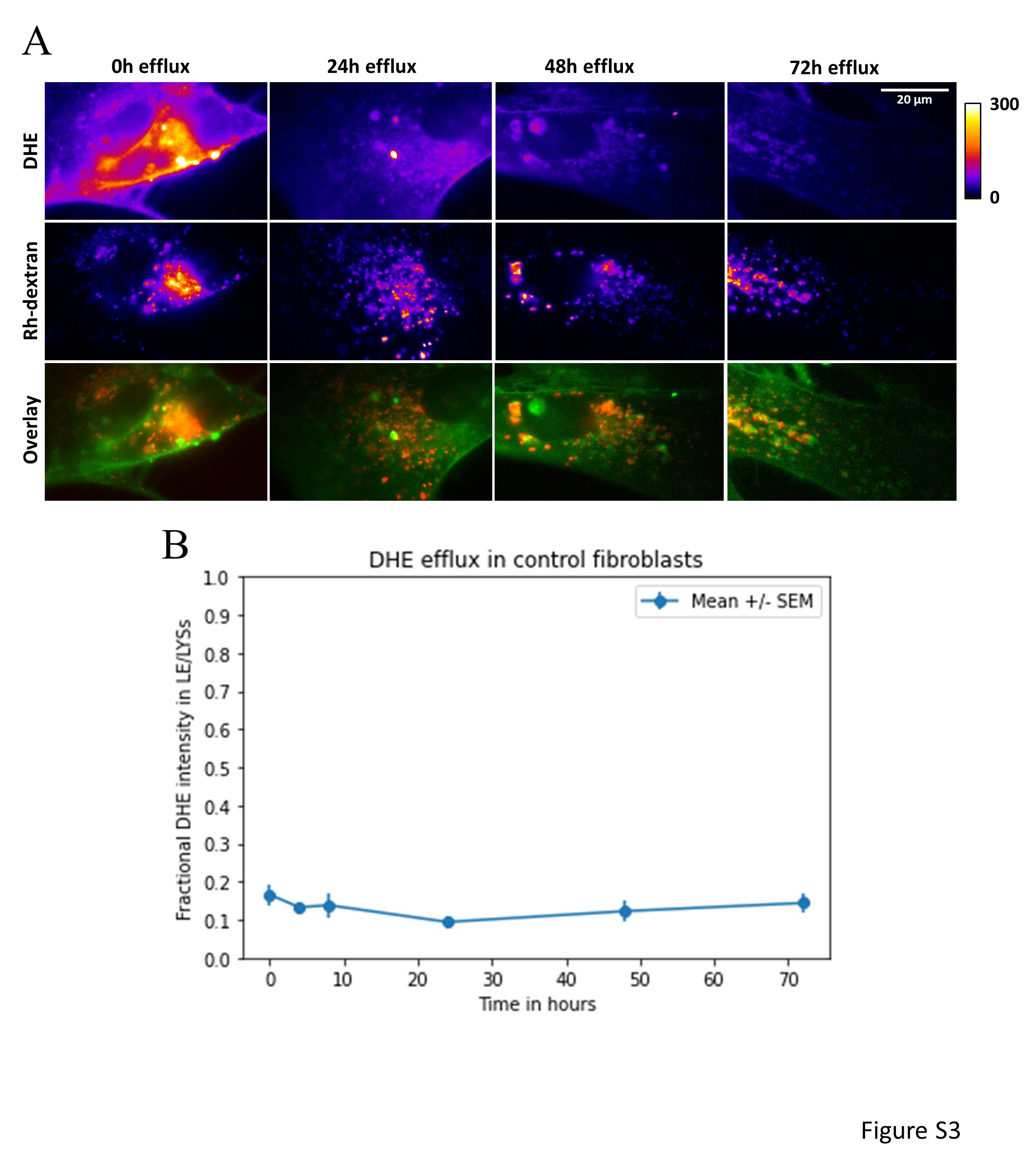

Supplement: Supplementary file 1 [file Image3.JPEG]

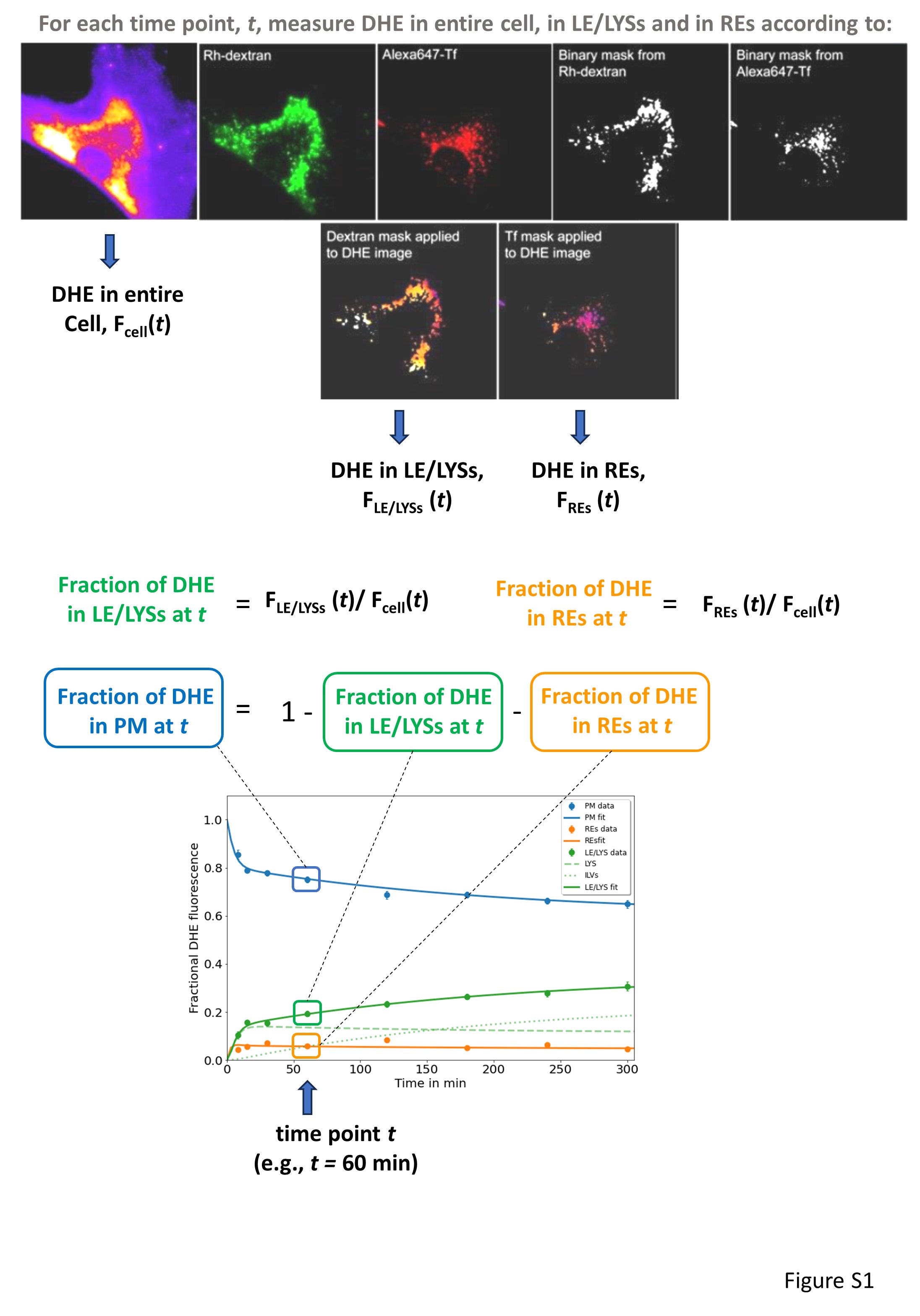

Supplement: Supplementary file 2 [file Image1.JPEG]

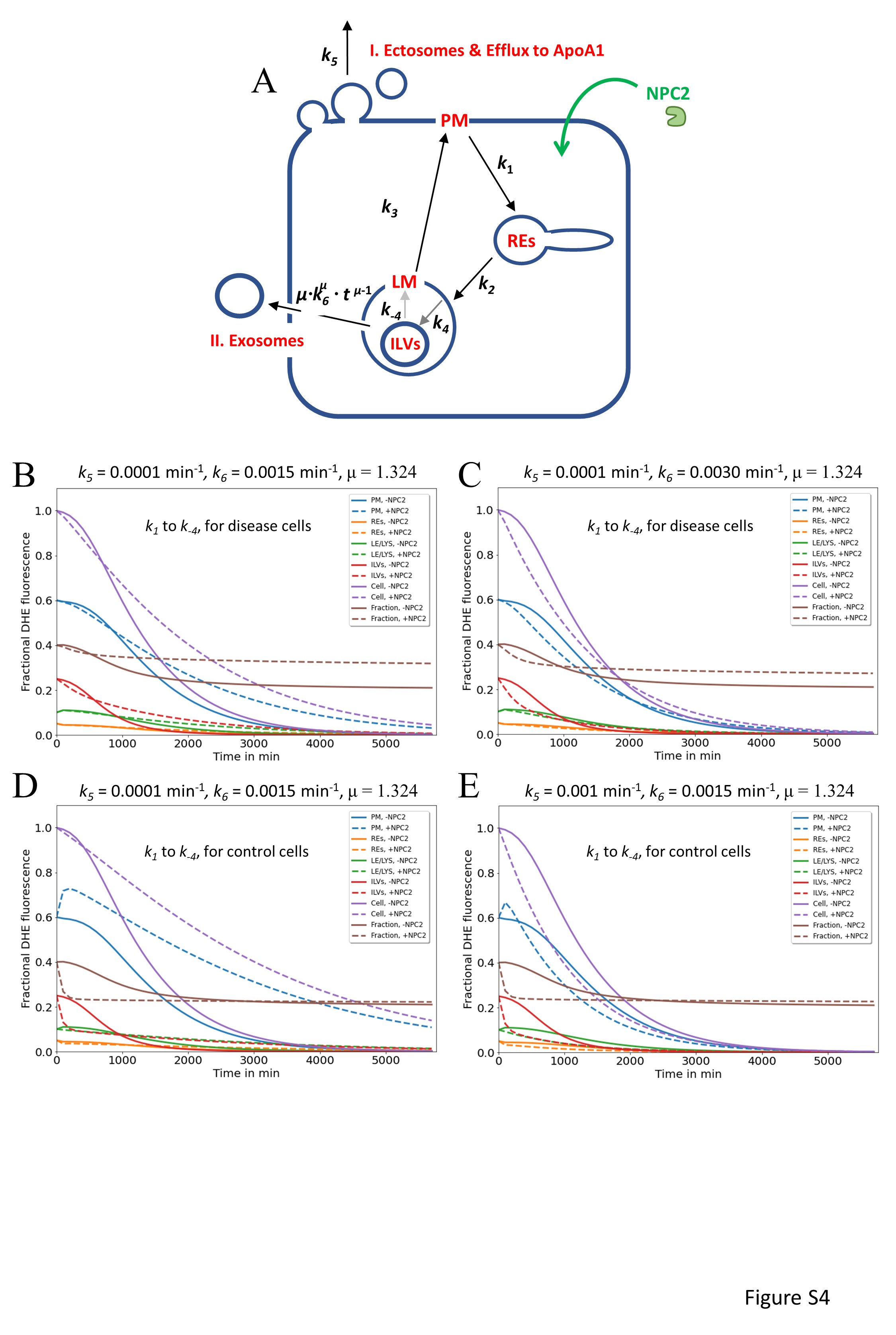

Supplement: Supplementary file 3 [file Image4.JPEG]

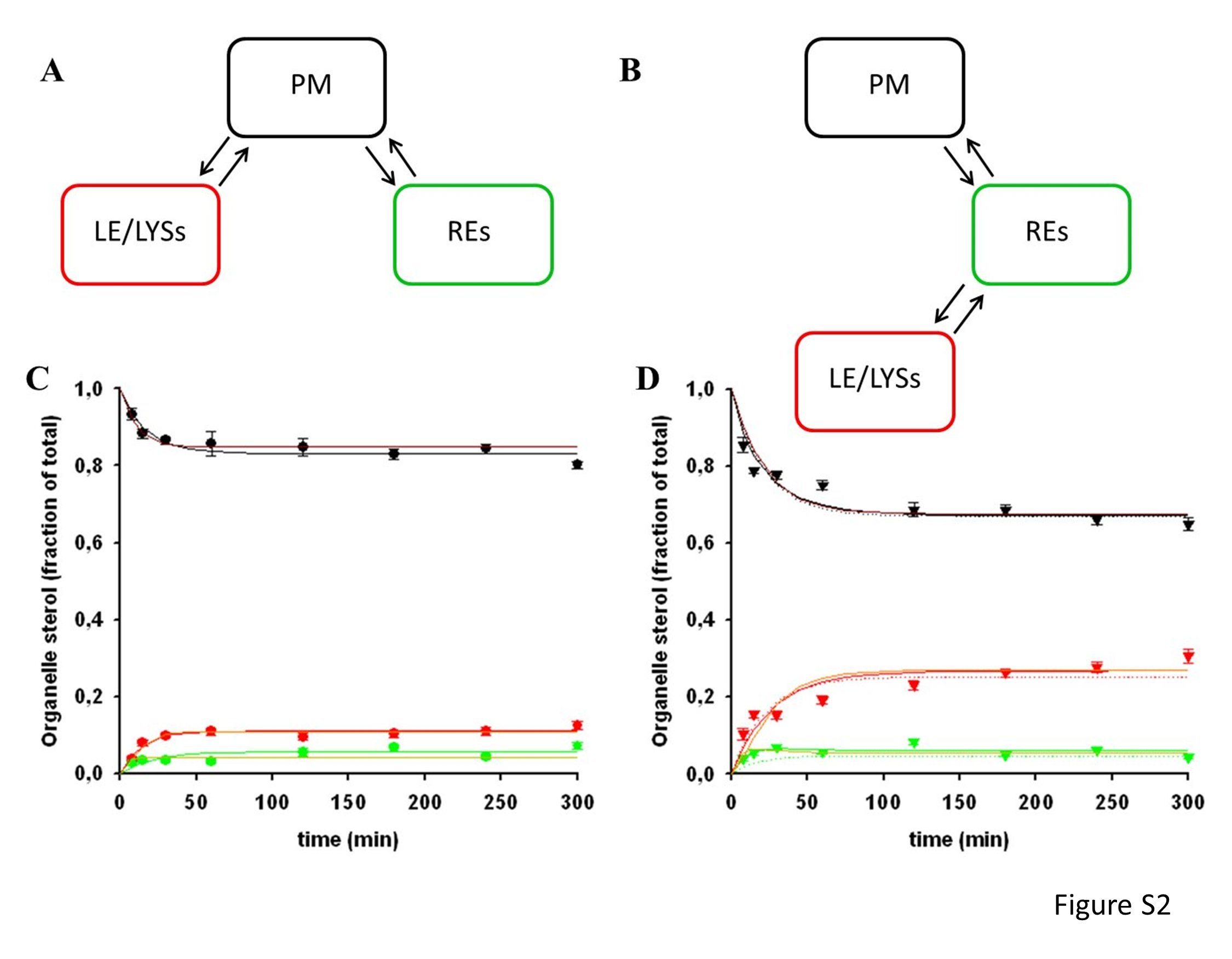

Supplement: Supplementary file 4 [file Image2.JPEG]

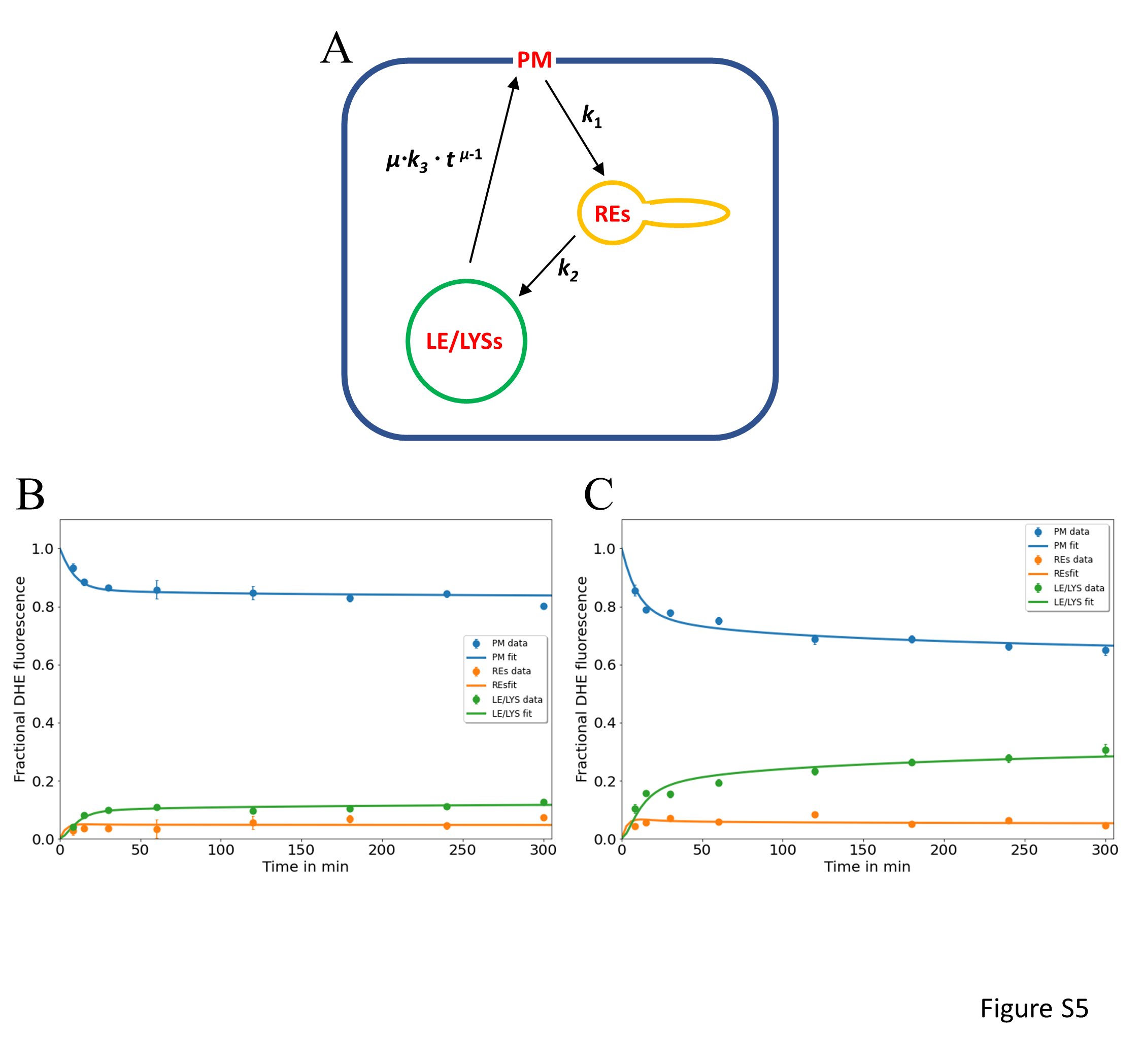

Supplement: Supplementary file 5 [file Image5.JPEG]
