# Supplementary material for: Kinetic modelling of sterol transport between plasma membrane and endo-lysosomes based on quantitative fluorescence and X-ray imaging data
Source: Front Cell Dev Biol. 2023 Oct 31;11:1144936. doi: 10.3389/fcell.2023.1144936 (PMC10644255; doi:10.3389/fcell.2023.1144936)
Supplement: Supplementary file 6 [file DataSheet1.PDF]

Supplementary materials

Steady state analysis of sterol fraction in subcellular compartments

Thus, the amount of DHE in each compartment at a steady state ($\overline{n_i}, \forall i = 1,\ldots,4$) can be analytically derived by setting Eqs 2 and 1b-d to zero and using linear algebra of inhomogeneous equation systems to obtain:

$$\overline{\boldsymbol{n_1}} = \frac{\boldsymbol{v_0}}{\boldsymbol{k_5}} = \boldsymbol{q_1} \qquad \overline{\boldsymbol{n_2}} = \frac{\boldsymbol{k_1 \cdot q_1}}{\boldsymbol{k_2}}; \qquad \overline{\boldsymbol{n_3}} = \frac{\boldsymbol{k_1 \cdot q_1}}{\boldsymbol{k_3}}; \qquad \overline{\boldsymbol{n_4}} = \frac{\boldsymbol{k_1 \cdot k_4 \cdot q_1}}{\boldsymbol{k_3 \cdot k_{-4}}} \qquad \text{(S1a-d)}$$

One can see that the steady state abundance in the PM ($\overline{n_1}$) is equal to the ratio of the inflow and release rate constants, and that the steady state sterol amount in all other compartments depends on this ratio as well, i.e., on $q_1 = v_0/k_5$, Since these parameters as well as their ratio are not known from the experiments, we seek expressions, that are independent of this ratio and at the same time measurable in experiments. These are the steady state fractions in each compartment **($\overline{\boldsymbol{n_i}}/\sum_{\boldsymbol{i=1\ldots4}}\overline{\boldsymbol{n_i}}$),** which are given by:

$$\frac{\overline{\boldsymbol{n_1}}}{\sum_{i=1\ldots4}\overline{\boldsymbol{n_i}}} = \frac{\boldsymbol{k_2 \cdot k_3 \cdot k_{-4}}}{\boldsymbol{s}}; \qquad \frac{\overline{\boldsymbol{n_2}}}{\sum_{i=1\ldots4}\overline{\boldsymbol{n_i}}} = \frac{\boldsymbol{k_1 \cdot k_3 \cdot k_{-4}}}{\boldsymbol{s}}; \qquad \frac{\overline{\boldsymbol{n_3}}}{\sum_{i=1\ldots4}\overline{\boldsymbol{n_i}}} = \frac{\boldsymbol{k_1 \cdot k_2 \cdot k_{-4}}}{\boldsymbol{s}};$$

$$\text{and } \frac{\overline{\boldsymbol{n_4}}}{\sum_{i=1\ldots4}\overline{\boldsymbol{n_i}}} = \frac{\boldsymbol{k_1 \cdot k_2 \cdot k_4}}{\boldsymbol{s}} \qquad \text{(S2a-d}$$

, where $\boldsymbol{s = k_2 \cdot k_3 \cdot k_{-4} + k_1 \cdot (k_3 \cdot k_{-4} + k_2 \cdot (k_4 + k_{-4}))}$**.** (S2e)

The steady state sterol fraction in the LE/LYSs corresponding to the sum of the fractions of n_3 and n_4 at steady state reads:

$$\frac{\overline{\boldsymbol{n_3}}}{\sum_{i=1\ldots4}\overline{\boldsymbol{n_i}}} + \frac{\overline{\boldsymbol{n_4}}}{\sum_{i=1\ldots4}\overline{\boldsymbol{n_i}}} = \frac{\boldsymbol{k_1 \cdot k_2 \cdot (k_4 + k_{-4})}}{\boldsymbol{s}} = \frac{\boldsymbol{k_1 \cdot k_2 \cdot (q_2 + 1)}}{\boldsymbol{k_2 \cdot k_3 + k_1 \cdot (k_3 + k_2 \cdot (q_2 + 1))}} \qquad \text{(S3)}$$

Here, the equilibrium constant between transport to and from ILVs is defined as $q_2 = k_4/k_{-4}$. Similarly, one can define the total intracellular sterol (ignoring minor non-determined fractions in the

ER and in mitochondria) by taking the sum of fractional fluorescence of DHE in LEs, ILVs and REs to obtain:

$$\frac{\overline{n_2}+\overline{n_3}+\overline{n_4}}{\sum_{i=1\ldots4}\overline{n_i}}=\frac{k_1\cdot k_3\cdot k_{-4}+k_1\cdot k_2\cdot(k_4+k_{-4})}{s}=\frac{k_1\cdot k_3+k_1\cdot k_2\cdot(q_2+1)}{k_2\cdot k_3+k_1\cdot(k_3+k_2\cdot(q_2+1))} \qquad \text{(S4)}$$

Importantly, the fractions, as determined in Eqs. 4-6 are independent of the influx/efflux ratio q_1. As inferred from Eq. 5 and 6, the sterol fraction in LE/LYSs (including ILVs; compartment 3 and 4) or in REs and LE/LYSs (compartment 2, 3 and 4) drops non-linearly as a function of the rate constant, k_{-4}. Thus, the higher the transport rate of sterol from ILVs back to the limiting membrane of LE/LYSs, the lower the overall sterol accumulation in cells. One can simplify these expressions by replacing the rate constants k_4 and k_{-4} by the equilibrium constant q_2 as in the right-hand side of Eq. 5 and 6. This gives the total intracellular DHE, i.e., the combined amount in REs and LE/LYSs, as shown in Eq. 6, which can be measured from images by intensity thresholding using the ImageJ plugin we presented previously (Berzina et al., 2018).

Estimation of sterol efflux efficiency in the absence of exosome release

Our model analysis predicts that the total amount of cholesterol delivered to cells in the continuous uptake experiment is higher in disease compared to control cells. This can be easily seen by summing Eqs. S1a-d to get the total amount of sterol delivered to cells from albumin at steady state:

$$\overline{n_1}+\overline{n_2}+\overline{n_3}+\overline{n_4}=\frac{q_1\cdot(k_1\cdot k_2(q_2+1)+k_1\cdot k_3+k_2\cdot k_3)}{k_2\cdot k_3} \qquad \text{(S5)}$$

Here, again we have $q_1=v_0/k_5$. By evaluating this expression for the parameter values of control and disease cells, respectively, one finds that the total cell-associated sterol delivered to cells is 4.5fold higher in disease compared to control cells. This is independent from the chosen ratio for the inflow rate and release rate constant from the PM (i.e., independent of q_1), as this parameter drops out when comparing the total sterol delivered to control and NPC2-deficient cells. W

Mathematical model of sterol efflux in the presence of exosome release

Including a time-dependent rate coefficient for sterol efflux from LE/LYSs via release of exosomes leads to the following modified equations for the PM (n_1) and the ILVs (n_4; compare Eqs. 1a, 1d and Eq. 4):

$$\frac{dn_1}{dt} = -(k_1 + k_5) \cdot n_1 + k_3 \cdot n_3 \qquad \text{(S6a)}$$

$$\frac{dn_4}{dt} = k_4 \cdot n_3 - (k_{-4} + \mu \cdot {k_6}^{\mu} \cdot t^{\mu-1}) \cdot n_4 \qquad \text{(S6b)}$$

This model was solved numerically as described in the main text.

To assess the impact of adding NPC2 protein to disease cells, we simulated sterol efflux also in rescued cells, i.e., in disease cells treated with NPC2 (Fig. S3A). By incubating DHE-loaded disease cells with purified NPC2 protein, we showed previously an enhancement of sterol efflux with less pronounced delay in endo-lysosomal sterol fraction compared to non-treated cells (Juhl et al., 2021). To simulate this scenario, the delay parameter was set to the value found previously for rescued cells (μ=1.324), and the rate constants for intracellular sterol transport (i.e., k_1 to k_{-4}) were set to those of disease cells (Fig. S3B and C) or of control cells (Fig. S3D and E). Only in the latter case, a pronounced drop in the endo-lysosomal sterol fraction was found compared to untreated cells (compare brown dashed and straight line in Fig. S3B to E). The experimentally observed rescue effect on sterol export from endo-lysosomes was milder than in these simulations, but this is due to the rather slow uptake of NPC2, which we found previously to have a half-time of 24h in disease fibroblasts (Lund et al., 2014). Thus, parameters will not change abruptly from disease to control cells but rather gradually under experimental conditions. Increasing additionally the rate constant for sterol release from the PM (i.e., k_5) prevents the transient sterol accumulation in the PM, supporting that sterol release from the PM via activation of ABCA1/ApoA1 can act in concert with NPC2-mediated

sterol mobilization from LE/LYSs in removing excess cholesterol from cells (Fig. S3D and E, blue and violet dashed lines).

Alternative mathematical description the pulse-chase experiment.

In an attempt to describe the kinetics of DHE transport to LE/LYSs from the pulse chase experiments without invoking a fourth compartment, an alternative kinetic description of intracellular sterol transport through the multi-vesicular endo-lysosomal compartment is described here. Such an alternative model might be motivated by delayed export of DHE from LE/LYSs back to the PM for example due to incomplete mixing of the cholesterol tracer with an endogenous immobile cholesterol pool. To account for this possibility, in describing sterol transport in human fibroblasts, a lag time model is put forward here as alternative to the four-compartment model. This lag-time model describes export of sterol from LE/LYS towards the PM by a rate coefficient, replacing the time-independent rate constant, k_3. This allows for coarse-graining the four-compartment model by lumping the description of endo-lysosomal membrane (compartment n_3) and ILVs (compartment n_4) into a new compartment n'_3, resulting in the following lag time compartment model:

$$\frac{dn_1}{dt} = -k_1 \cdot n_1 + k_3 \cdot t^{-\alpha} \cdot n'_3$$

$$\frac{dn_2}{dt} = k_1 \cdot n_1 - k_2 \cdot n_2$$

$$\frac{dn_3}{dt} = k_2 \cdot n_2 - k'_3 \cdot t^{-\alpha} \cdot n_3 \qquad \text{(S7a-c)}$$

The new time dependent rate coefficient $k'_3 \cdot t^{-\alpha}$ with anomaly parameter α accounts for the delayed export of sterol from the endo-lysosomal compartment. The parameter $\mu = 1-\alpha$ is the stretching exponent defined in the main text (see Eq. 8) and describes for $\mu<1$, corresponding to $\alpha > 0$ a stretched exponential decay, which is faster than exponential for short times but slower with a

power-law behavior for long times (Macheras and Iliadis, 2006). By setting the rate constant $k_3' = \mu \cdot k_3^{\mu}$ and $t^{-\alpha} = t^{\mu-1}$ one recovers an identical expression as for the delay term in Eq. 10b of the main text. This expression is used here to model heterogeneous export of DHE from LE/LYSs back to the PM. The corresponding survival time for sterol in endo-lysosomes, i.e., lysosomal sterol exit kinetics is proportional to $\exp(-[k'_3/(1-\alpha)]\cdot t^{1-\alpha})$ and thereby follows a stretched exponential decay for $\alpha > 0$ (Macheras and Iliadis, 2006;Dokoumetzidis and Macheras, 2009). This is readily shown by integrating the second term on the right-hand side of Eq. S1c for an initial amount in compartment n_3 corresponding to its equilibrium value, which gives an expression equivalent to Eq. 8 of the main text. For $\alpha = 0$, the lag time model is equal to a simple three-compartment model with a time-independent rate constant, $k'_3 = k_3$ for sterol transport between LE/LYS and PM, as before. We fit this model first to the data of DHE transport in control cells and obtain the parameter values shown in Tab. S1. The value for the anomaly parameter is $\alpha = 0.0075 \pm 0.0351$ which gives $\mu = 0.9925 \approx 1$, being characteristic for mono-exponential sterol export from LE/LYSs in control cells. This is in line with the model analysis using the three-compartment model for control cells. Next, we employed this lag-time model to the transport data of DHE in disease cells, in which we kept the rate constants for sterol transport between LE/LYSs and PM, k'_3, fixed to the value determined in control cells. All other parameters, i.e., k_1, k_2 and the new parameter α were allowed to vary. This resulted in a very good fit of the delay model to the kinetic data for control and disease cells (Fig. S4). The value for the anomaly parameter was $\alpha = 0.1906 \pm 0.0321$ for NPC2-deficient cells, corresponding to $\mu = 0.8094$, being characteristic for a stretched exponential export kinetics from LE/LYSs. Such kinetic properties are characteristic for delayed transport processes with power-law behavior for long times (Wise, 1985;Norwich, 1997;Macheras and Iliadis, 2006). However, $\mu<1$ is not in accordance with our efflux experiments, where we found $\mu>1$, corresponding to a positive

power law which is characteristic for an initial delay with increased efflux speed over time, i.e., a compressed exponential kinetic behavior.

Table S1: Parameters for DHE transport according to the lag-time multi-compartment model

Time courses of DHE transport from the PM to REs and LE/LYSs in control cells were fitted to the multi-compartment lag-time model given in Eq. S1. Parameters values were optimized in parallel to all compartments by non-linear regression. The mean value, standard deviation (±) and coefficient of variation (CV) of estimated parameters is given, as provided by the SYMFIT software.

**Rate constant**	k_1 (min^{-1}) (PM→REs)	k_2 (min^{-1}) (REs→LE/LYSs)	k'_3 (min^{-1}) (LE/LYSs→PM)	α (Anomaly exponent)
**Control cells**	0.0137 ± 0.0016 CV=0.1182	0.2458 ± 0.0329 CV=0.1340	0.1496 ± 0.0389 CV=0.2601	0.0724 ± 0.0351 CV=0.4843
**Disease cells**	0.0216 ± 0.0021 CV=0.0751	0.2733 ± 0.0282 CV=0.0084	0.1496* *fixed	0.1906 ± 0.0321 CV=0.1682

Additional references

Berzina, Z., Solanko, L.M., Mehadi, A.S., Jensen, M.L.V., Lund, F.W., Modzel, M., Szomek, M., Solanko, K.A., Dupont, A., Nielsen, G.K., Heegaard, C.W., Ejsing, C.S., and Wustner, D. (2018). Niemann-Pick C2 protein regulates sterol transport between plasma membrane and late endosomes in human fibroblasts. *Chem Phys Lipids* 213**,** 48-61.

Dokoumetzidis, A., and Macheras, P. (2009). Fractional kinetics in drug absorption and disposition processes. *J Pharmacokinet Pharmacodyn* 36**,** 165-178.

Juhl, A.D., Lund, F.W., Jensen, M.L.V., Szomek, M., Heegaard, C.W., Guttmann, P., Werner, S., Mcnally, J., Schneider, G., Kapishnikov, S., and Wüstner, D. (2021). Niemann Pick C2 protein enables cholesterol transfer from endo-lysosomes to the plasma membrane for efflux by shedding of extracellular vesicles. *Chem. Phys. Lipids* 235**,** 105047.

Lund, F.W., Jensen, M.L.V., Christensen, T., Nielsen, G.K., Heegaard, C.W., and Wüstner, D. (2014). SpatTrack, an imaging toolbox for analysis of endosome motility and distribution. *Traffic* 15**,** 1406-1429.

Macheras, P., and Iliadis, A. (eds.). (2006). *Modeling in Biopharmaceutics, Pharmacokinetics, and Pharmacodynamics.* New Yrok: Springer.

Norwich, K.H. (1997). Noncompartmental models of whole-body clearance of tracers: a review. *Ann Biomed Eng* 25**,** 421-439.

Wise, M.E. (1985). Negative power functions of time in pharmacokinetics and their implications. *J Pharmacokinet Biopharm* 13**,** 309-346.
